# Inhaled furosemide for relief of air hunger versus sense of breathing effort: a randomized controlled trial

**DOI:** 10.1186/s12931-018-0886-9

**Published:** 2018-09-20

**Authors:** Joanna C. Grogono, Clare Butler, Hooshang Izadi, Shakeeb H. Moosavi

**Affiliations:** 1 Department of Health and Life Sciences, Oxford Brookes University, Gipsy Lane Campus, Headington, Oxford, OX3 0BP UK; 2 Department of Nursing, Oxford Brookes University, Marston Road Site, Oxford, OX3 0FL UK; 3 School of Engineering, Computing and Mathematics, Oxford Brookes University, Wheatley Campus, Wheatley, Oxford, OX33 1HX UK

**Keywords:** Aerosolized, Loop diuretics, Hypercapnia, Resistive load, Dyspnoea, Nebuliser, Breathlessness, Pulmonary stretch receptors

## Abstract

**Background:**

Inhaled furosemide offers a potentially novel treatment for dyspnoea, which may reflect modulation of pulmonary stretch receptor feedback to the brain. Specificity of relief is unclear because different neural pathways may account for different components of clinical dyspnoea. Our objective was to evaluate if inhaled furosemide relieves the air hunger component (uncomfortable urge to breathe) but not the sense of breathing work/effort of dyspnoea.

**Methods:**

A randomised, double blind, placebo-controlled crossover trial in 16 healthy volunteers studied in a university research laboratory. Each participant received 3 mist inhalations (either 40 mg furosemide or 4 ml saline) separated by 30–60 min on 2 test days. Each participant was randomised to mist order ‘furosemide-saline-furosemide’ (n- = 8) or ‘saline-furosemide-saline’ (*n* = 8) on both days. One day involved hypercapnic air hunger tests (mean ± SD PCO_2_ = 50 ± 3.7 mmHg; constrained ventilation = 9 ± 1.5 L/min), the other involved work/effort tests with targeted ventilation (17 ± 3.1 L/min) and external resistive load (20cmH_2_O/L/s). Primary outcome was ratings of air hunger or work/effort every 15 s on a visual analogue scale. During saline inhalations, 1.5 mg furosemide was infused intravenously to match the expected systemic absorption from the lungs when furosemide is inhaled. Corresponding infusions of saline during furosemide inhalations maintained procedural blinding. Average visual analogue scale ratings (%full scale) during the last minute of air hunger or work/effort stimuli were analysed using Linear Mixed Methods.

**Results:**

Data from all 16 participants were analysed. Inhaled furosemide relative to inhaled saline significantly improved visual analogues scale ratings of air hunger (Least Squares Mean ± SE − 9.7 ± 2%; *p* = 0.0015) but not work/effort (+ 1.6 ± 2%; *p* = 0.903). There were no significant adverse events.

**Conclusions:**

Inhaled furosemide was effective at relieving laboratory induced air hunger but not work/effort in healthy adults; this is consistent with the notion that modulation of pulmonary stretch receptor feedback by inhaled furosemide leads to dyspnoea relief that is specific to air hunger, the most unpleasant quality of dyspnoea.

**Funding:**

Oxford Brookes University Central Research Fund.

**Trial registration:**

ClinicalTrials.gov Identifier: NCT02881866. Retrospectively registered on 29th August 2018.

## Background

Dyspnoea accounts for over 15% of symptom burden among hospitalized patients and contributes to poor quality of life by limiting activity, increasing anxiety levels and undermining the will to live [1, 2]. It is present in a wide range of conditions such as chronic obstructive pulmonary disease (COPD), chronic heart failure, advanced cancer and neuromuscular disease [3]. Given its prevalence and impact, there is an urgent clinical need for more effective treatments. Inhaled furosemide offers a potential complementary treatment for dyspnoea relief [4].

Furosemide is a loop diuretic. It is usually taken orally or intravenously and acts through inhibition of the sodium-potassium-chloride co-transporter in the thick ascending limb of the loop of Henle in the kidneys [5]. In rats, inhaled furosemide has been shown to sensitize slowly adapting pulmonary stretch receptors (saPSR) in the lung parenchyma [6]. Stimulation of these receptors has been shown to relieve air hunger (AH; an uncomfortable urge to breathe) in high level quadriplegic humans in whom afferent information from the chest wall is blocked but vagal afferents from lungs remain intact [7]. Along with AH, clinical dyspnoea is comprised of other distinguishable components including the sense of breathing work/effort (WE) and chest ‘tightness’ [8]. These components can vary based on interactions between physiological, psychological, social and environmental factors [9]. The mechanisms underlying dyspnoea are complex with multiple voluntary and involuntary triggers as well as feed-forward and feed-back mechanisms [9]. Measuring breathlessness is difficult as the sensation of breathlessness is subjective and does not correlate well with objective measures of lung or heart function [10, 11]. The distinct components of clinical dyspnoea are thought to arise from separate neural pathways [8]. This theory comes from studies showing that despite complete paralysis of the respiratory muscles, subjects show the same AH stimulus-response to CO_2_ and that increasing the tidal volume using a ventilator can relieve AH in C1-C2 quadriplegics, suggesting a vagal pathway rather than feedback from chest wall afferents [7, 12, 13]. For AH a collorary discharge of the drive to breathe from the brain stem has been proposed [14] whereas for WE a corollary discharge from the motor cortex driving voluntary breathing has been proposed as the source of the sensation [12].

The optimal solution for relief of dyspnoea is to treat the underlying pathology but this is not always possible and does not always lead to symptom relief. In chronic conditions, such as heart failure or COPD, symptom control becomes a priority in order to improve quality of life. A newer focus is to alter the perception of dyspnoea via altering the activity of neural signals sent to the brain reporting the prevailing level of breathing. The mechanism of action of inhaled furosemide has not been fully elucidated but current theory suggests that it acts by modulating pulmonary stretch receptor activity. There is evidence to support this theory, both in animal and human studies [6, 15].

The current study hypothesized that inhaled furosemide would relieve AH but not the sense of breathing effort. The result of this study has been previously reported in the form of an abstract [16].

## Methods

Sixteen healthy volunteers (9 male) attended the Oxford Brookes Cardiorespiratory Research Laboratory on 4 occasions. Eligibility criteria included; age above 18 years, no regular prescription medication in the previous 2 weeks and if female, not pregnant or planning pregnancy. Oxford Brookes University Research Ethics committee approved the protocol and all participants provided written informed consent.

All participants and all healthcare professionals apart from those who administered the interventions were blinded to the medications. Each participant visited the laboratory on 4 occasions; two practice sessions to familiarise themselves with the equipment and to become accustomed to rating the sensation of dyspnoea and; two ‘test’ sessions where participants inhaled the mists, with different dyspnoea stimuli (AH or WE) on different days in random order. On these days the participants were randomised to either inhale aerosolized mist (nebuliser) in the order of furosemide (40 mg, 10 mg/ml; hameln pharmaceuticals gmbh, Langes Feld, Hameln, Germany), saline (4 ml; B.Braun, Melsungen, Germany), furosemide (FSF) or saline, furosemide, saline (SFS) for both study days. Prior to each mist inhalation they gargled with a menthol mouthwash. The nebulisation duration of the furosemide mist was approximately 10-15 min and the saline mist 5-10 min. Each mist inhalation started after 6–11 min of the steady state test level of each pre-mist AH or WE test. The post mist steady state test level was between 9 and 14 min after the end of the mist inhalations. Each AH or WE test lasted 10 min, with a total visit duration of around 3 h (7 AH or WE tests, and 3 mist inhalations.)

### Dyspnoea stimuli

Two different dyspnoea stimuli were tested in each volunteer on different days.

1. Air Hunger (AH).

**Fig. 1:**
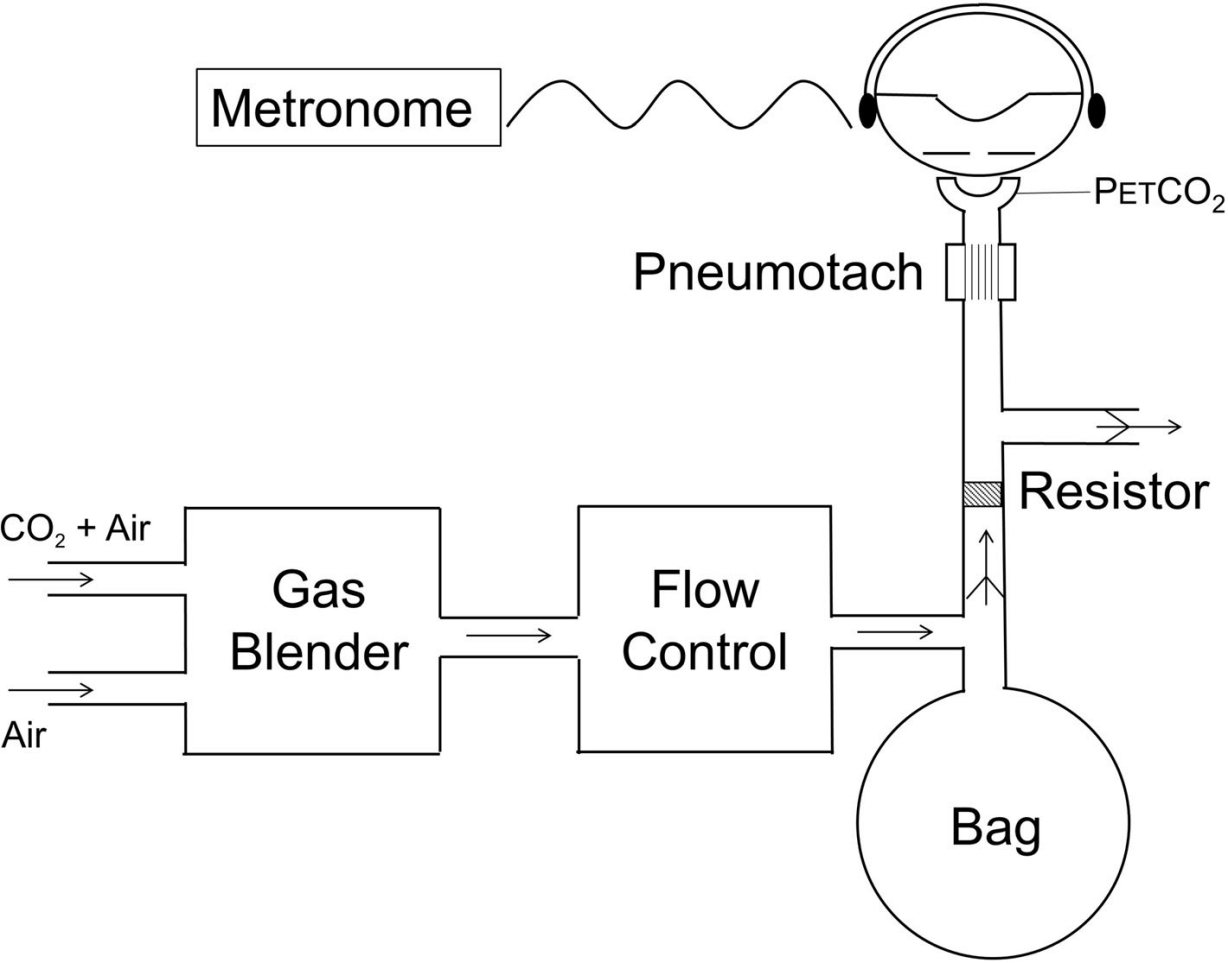
Breathing circuit. The Breathing Circuit was identical for air hunger (AH) test and work/effort (WE) test, except that the external resistance was removed in the AH test. To elicit AH, CO_2_ was added to the flow of fresh gas into the bag and this flow was fixed at baseline alveolar ventilation. To elicit WE, individuals were instructed to empty the bag with each breath while the flow of fresh bag into the bag was increased and CO_2_ was added to maintain normocapnia.  P_ET_CO_2_ = end tidal PCO_2_

**Fig. 2:**
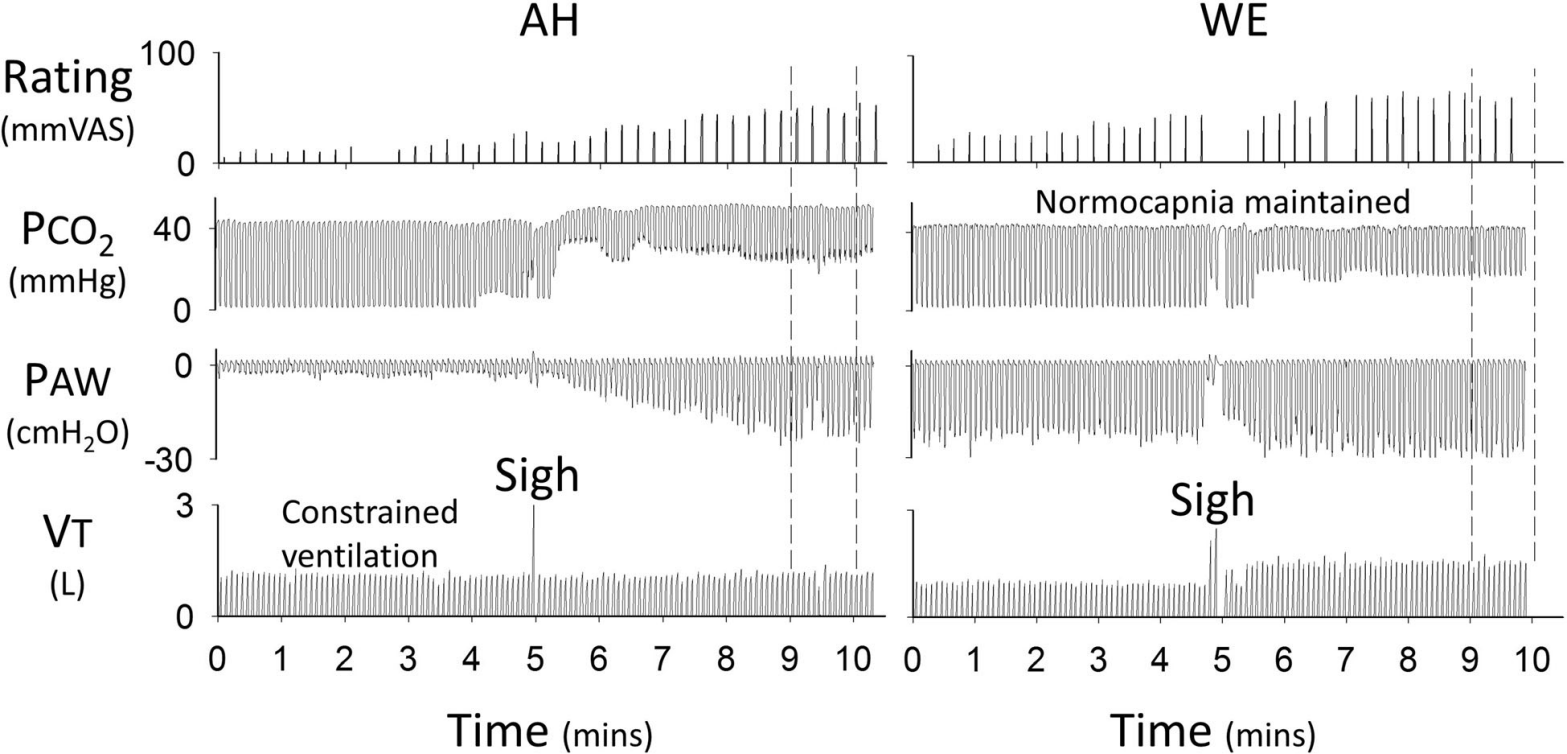
Standard tests of air hunger and work/effort. Left: Typical raw data set for the air hunger (AH) test during which two levels of end-tidal PCO_2_ were imposed and ventilation was constrained. The vertical dashed lines indicate the steady state level of AH associated with the test level of CO_2_ chosen to elicit 50%VAS ratings in pre-mist trials. Right: Typical raw data set for the work effort (WE) test in which two levels of targeted VT were imposed and normocapnia was maintained. The vertical dashed lines indicate the steady state level of WE associated with the test level of VT chosen to elicit 50% VAS ratings in pre-mist trials. During both tests ventilatory constraint or targeting was suspended briefly and participants were instructed to take a sigh. VAS ratings were provided every 15 s in response to a LED cue. *VT* Tidal volume, *PAW* continuous airway pressure measured at the mouth

Participants were semi-reclined in a padded chair, whilst wearing a nose clip, and breathing via a mouthpiece. Humidified warmed gas was delivered into a 3 l anaesthetic bag supplying the inspiratory gas via a one-way valve. Expired gas was expelled via a second one-way valve (Fig. 1). Minute ventilation was therefore constrained as it could not exceed the flow into the bag. The participant’s respiratory rate was fixed by breathing in time with a metronome. The frequency and tidal volume were therefore fixed. Participants were informed that the amount of air at times would be limited and were coached not to pull excessive pressure with ineffective efforts against the collapsed bag. During the study carbon dioxide was added to the inspired air. Initially a gradual increase in inspired CO_2_ was performed until the maximal tolerated level of dyspnoea and then two 5 min steady state levels of end tidal CO_2_ (ETCO_2_) were chosen to target a level of 50% (‘test’ level) and 25% (‘masking’ level) of the visual analogue scale (VAS) for AH. The ‘masking’ level served to prevent the participants from expecting a certain result. The order of the 5 min test and masking steps were altered between runs. This method has been shown to produce strong AH stimulation without significant WE sensation [17]. Brief periods of unrestrained breathing separated the two levels of hypercapnia during which participants performed an inspiratory capacity manoeuvre in order to facilitate rapid change in inspired CO_2_ level and to reduce the chance of atelectasis (Fig. 2).

2) Work Effort (WE)

Participants were semi-reclined in a padded chair, whilst wearing a nose clip, and breathing via a mouthpiece. Humidified warmed gas was delivered into a 3 l anaesthetic bag supplying the inspiratory gas via a one-way valve. Two resistors (12cmH_2_O and 8cmH_2_O at 1 L/s in series) were added to the inspiratory side of the circuit, giving an estimated total resistance of 20cmH_2_O as it has been shown that resistances of series combination are approximately equivalent to the algebraic sum of the individual resistors [18]. Expired gas was expelled via a second one-way valve. The participant was instructed to just empty the anaesthetic bag with each breath and a metronome fixed the frequency of each breath. Therefore, the amount of gas flowing into the bag determined the targeted minute ventilation. This target flow began at a level that matched the individual’s baseline alveolar ventilation and then was gradually increased until the participant could no longer empty the bag (or a maximum of 20 l/min –the limit of the flowmeter device). This was followed by two 5-min steady state levels of targeted ventilation, a ‘test’ level that generated 50% WE on the visual analogue scale and a ‘masking’ level generating 25%. These two steady state levels were imposed in random order to prevent the participants from expecting a certain result. This stimulus was always limited by participants failing to meet a higher ventilation target and not because they reached the top of the VAS for WE. Normocapnia (mean ± SD: 41.9 ± 1.2 mmHg) was maintained throughout. Brief periods of unrestrained breathing separated the two levels of ventilation during which participants performed an inspiratory capacity manoeuvre in order to match the design of the AH test (Fig. 2).

### Intravenous infusions

During each inhalation period, participants also received a 15-min (1 ml/min) intravenous infusion of 0.1 mg/ml solution furosemide if inhaled substance was 0.9% sodium chloride, or 0.9% sodium chloride if inhaled substance was furosemide (i.e. SFS infusions for FSF inhalations versus FSF infusions for SFS inhalations.) Infusions were administered via a catheter inserted in the antecubital vein using a syringe driver (Graseby In-line Pressure Syringe Pump 3200, Graseby Medical Ltd., Ashford, UK). 1.5 mg furosemide was infused over a period of time set to match the duration of inhalation. This dose was chosen as this was the amount expected to be systemically absorbed from a 40 mg nebuliser dose [19]. This ensured that both participants and the researchers did not know which mist was furosemide or 0.9% sodium chloride since the systemic effects (diuresis) were similar in each case. It also allowed an assessment of the systemic effect on furosemide on dyspnoea relief.

### Measurements

A 100 mm electronic VAS was used to obtain subjective ratings of both AH (during AH tests) and WE (during WE tests). Ratings were cued by a ‘rate now’ light every 15 s, which instructed participants to rate how much AH or WE they were feeling at that point in time, throughout the 10 mins of each AH and WE test. In the practice sessions, participants were immediately asked to select descriptors from a set list for any of the sensations they felt during that test. In future test sessions they were asked to focus on those descriptors that matched the AH sensation for the AH test and WE sensation for the WE test. The range covered 0 mm (no breathlessness) to 100 mm (tolerable limit) as previously described [17, 20, 21]. Additional word anchors (‘slight’, ‘moderate’ and ‘severe’) were placed at equal separation alongside the scale, which enabled participants to remember how much of the scale represented how much sensation from one occasion to the next. The order of test sessions (AH or WE) were randomly allocated and counterbalanced.

Airflow was measured via a pneumotachometer (Respiratory Flow Head MLT300L, ADinstruments, Oxford, UK) and integrated (FV156 respiratory flow integrator, Validyne Engineering, CA, USA) to provide tidal volume (VT). Breathing pattern was recorded by DC-coupled respiratory inductance plethysmography ((RespiTrace R250, Studley Data Systems, Oxford, UK). Mouthpiece pressure was measured via a fine-bore (1.5 mm) sampling tube inserted into the mouthpiece connected to a pressure transducer (Differential pressure transducer, ±50 cmH20, Validyne Engineering, CA, USA), Tidal PCO_2_ and PO_2_ were measured with a calibrated, fast-responding, respiratory gas analyser (ML206, ADinstruments, Oxford, UK). Blood pressure, oxygen saturations (SaO_2_) and electrocardiogram were also monitored (DatexOhmeda Cardiocap 5, Madison USA). Signals were digitalised and recorded for offline analysis (Micro1401 with Spike 2 software, Cambridge Electronic Design, Cambridge, UK.)

Participants voided prior to the start of each test session and the output was measured at approximately 25 min after each mist inhalation by urinating into a measuring flask.

### Data analysis

**Fig. 3:**
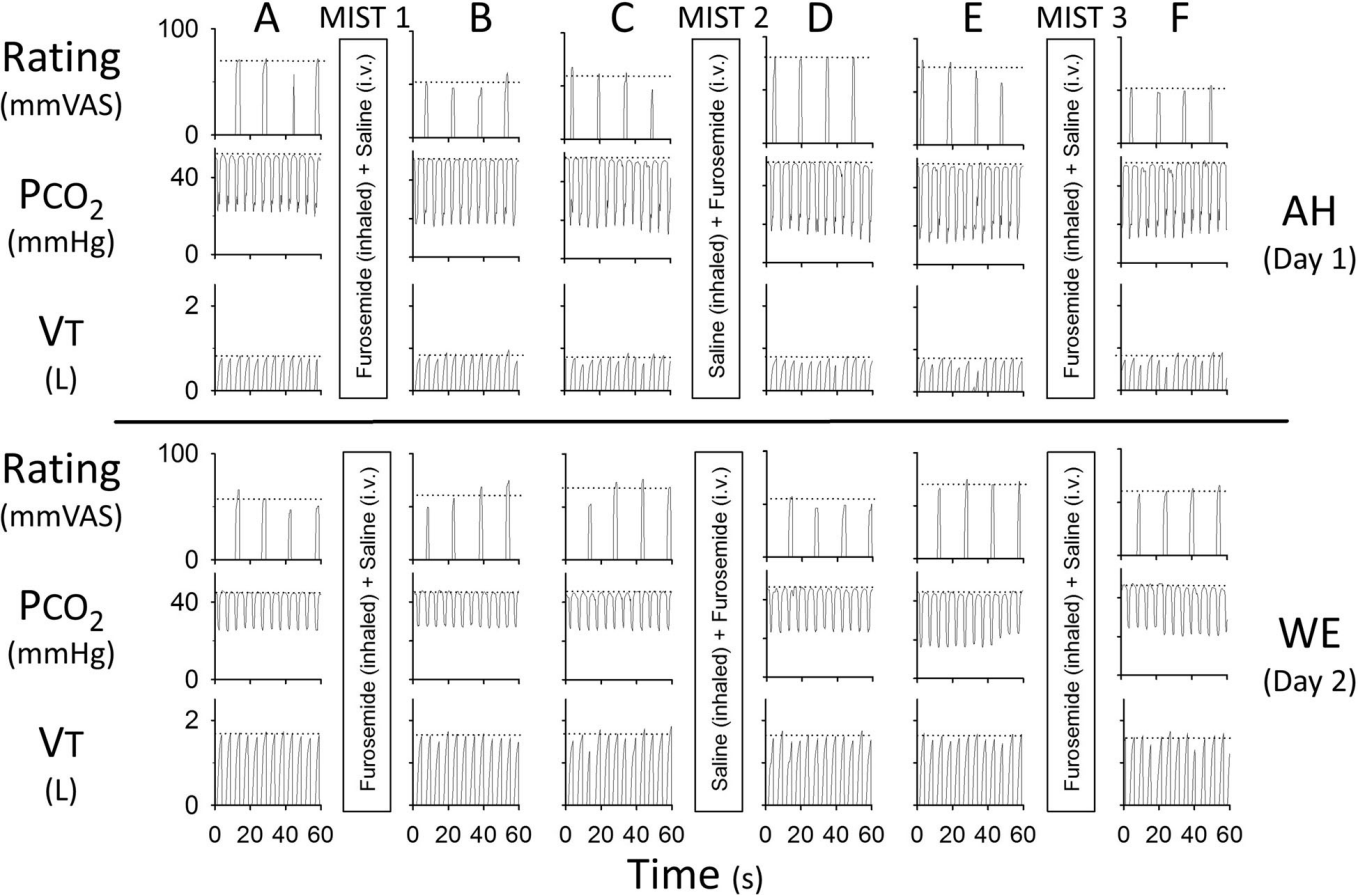
Effect of mist inhalations on steady state air hunger (AH) and work/effort (WE). This dataset is from an individual who received the mists in the order of furosemide-saline-furosemide (FSF) with the corresponding saline-furosemide-saline (SFS) intravenous infusions on both days. Panels **a** to **f** show the last minute of each test level of end-tidal CO_2_ (for AH) or of VT (for WE) –these regions of interest are shown by the vertical dashed lines in Fig. 2. AH test day: Air hunger ratings were reduced after furosemide inhalation (**a** to **b** and **e** to **f**) but not after saline inhalation (**c** to **d**). WE test day: No obvious differences in ratings were evident before and after any mist inhalations

The VAS in the last minute of each test step for the two furosemide mists presented for half the participants were averaged (those in the FSF group) and for the 2 saline mists presented for the other half (for those in the SFS group). The Linear Mixed Model “mixed” procedure of SAS 9.4 was used to analyse the data. Initially a full mean model with three factors; two levels of ‘condition’ (AH or WE), two levels of ‘mist’ (Furosemide or Saline), and 7 levels of ‘time’ (tests A-G; Fig. 3). All the 2-way and 3-way interactions were examined. Reducing the mean model by removing non-significant terms individually, resulted in the final model with 3 main effects and one interaction between condition-mist.

### Sample size

In a preliminary study 10 healthy volunteers rated 13% lower AH on VAS with inhaled furosemide relative to inhaled saline [21], with a standard deviation of 16% resulting in an effect size of 0.81. Based on this observation, it was determined that 16 participants were required to detect an effect size of 0.81 using matched pairs t-test at 5% significance level and 86% power.

### Randomisation

**Fig. 4:**
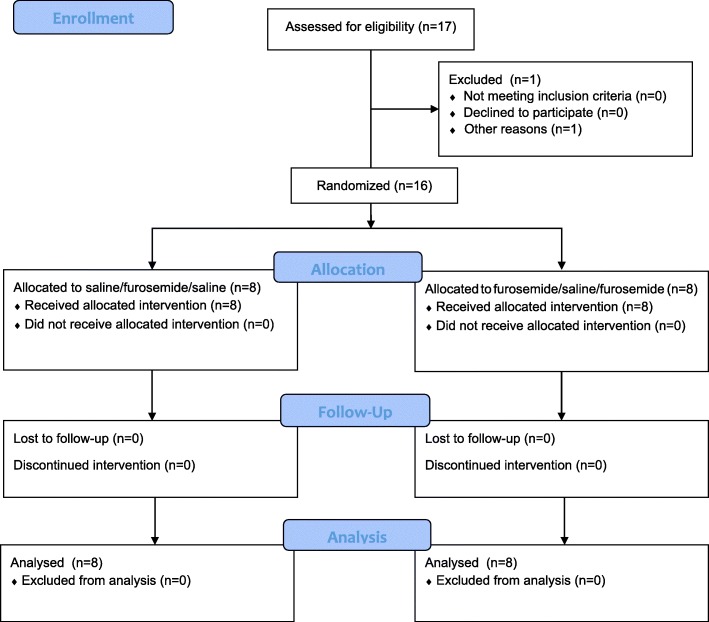
Patient Flow Diagram

Participants were randomised to mist allocations after completing the two practice sessions. One of 17 recruited participants did not progress to randomisation (Fig. 4); this was because despite increasing levels of hypercapnia (up to PETCO_2_ of 54 mmHg), they rated near zero dyspnoea and self-terminated the test due to light-headedness during practice sessions. When questioned they denied any experience of dyspnoea during the test.

The mist order allocation (FSF or SFS) was randomly assigned by the unblinded researcher to 16 sequential numbers, ensuring that 8 participants were allocated to the SFS group and 8 to the FSF group. A blinded researcher assigned each consecutive participant to the next available allocation number. Apart from the unblinded researcher, all other investigators and participants did not know whether the allocated number corresponded to FSF or SFS group. Once full analysis had been completed the principal investigator was provided with the allocation code.

Participants were provided with detailed written information about the interventions and protocol. They were aware they were going to receive furosemide or a placebo (control substance, saline) to inhale as a mist and to have as a solution via a vein in the arm (that was not the same as the mist) 3 times per visit. The diagram in the patient information leaflet showed the mist order as A, B, A.

## Results

Participants were recruited between 1st October 2015, and the first participant enrolled on 6th October 2015 and the last participant was enrolled on 26nd February 2016. The last visit for the last participant was on 11th March 2016. The median duration for all visits was 19 days.

The baseline characteristics of the participants who completed the study are shown in Table 1. The FSF mist order group and the SFS mist order group were well matched apart from by chance a higher proportion of participants who were Caucasian in the FSF compared to the SFS groups (*p* = 0.031). It is notable that 2 of the 3 (S9, S12) individuals who had an increase in AH (rather than a relief) following inhaled furosemide had a history of asthma. These two and S15 who also had a history of asthma were in the SFS group. No other notable differences were observed for individuals with a history of asthma.

**Table 1:** Participant Characteristics

	Total	FSF	SFS	*p* value
Number	16	8	8	NS
Males: Female	9: 7	4: 4	5: 3	NS
Mean age, yr. (mean ± SD)	24.3 ± 3.7	23.6 ± 3.1	25 ± 4.3	NS
Caucasian: Non-Caucasian	11: 5	8: 0	3: 5	*0.031
Mean height, m (mean ± SD)	1.7 ± 0.1	1.7 ± 0.1	1.7 ± 0.1	NS
Mean weight, kg (mean ± SD)	79.5 ± 24	88.4 ± 28	70.6 ± 17	NS
History of Asthma	3	0	3	NS
Smoker/Ex-smoker: Never smoked	5: 11	3: 5	2: 6	NS
Previous experience with breathing apparatus	11	6	5	NS
Regular Sport: Sedentary	14: 2	7: 1	7: 1	NS

*Abbreviations: FSF* Mist order inhalation furosemide-saline-furosemide, *SFS* Mist order inhalation saline-furosemide-saline, *NS* not significant

*Note*: Experience of breathing apparatus included snorkelling, scuba diving or previous testing

*p < 0.05

### Effect of mist inhalations on AH and WE

**Fig. 5:**
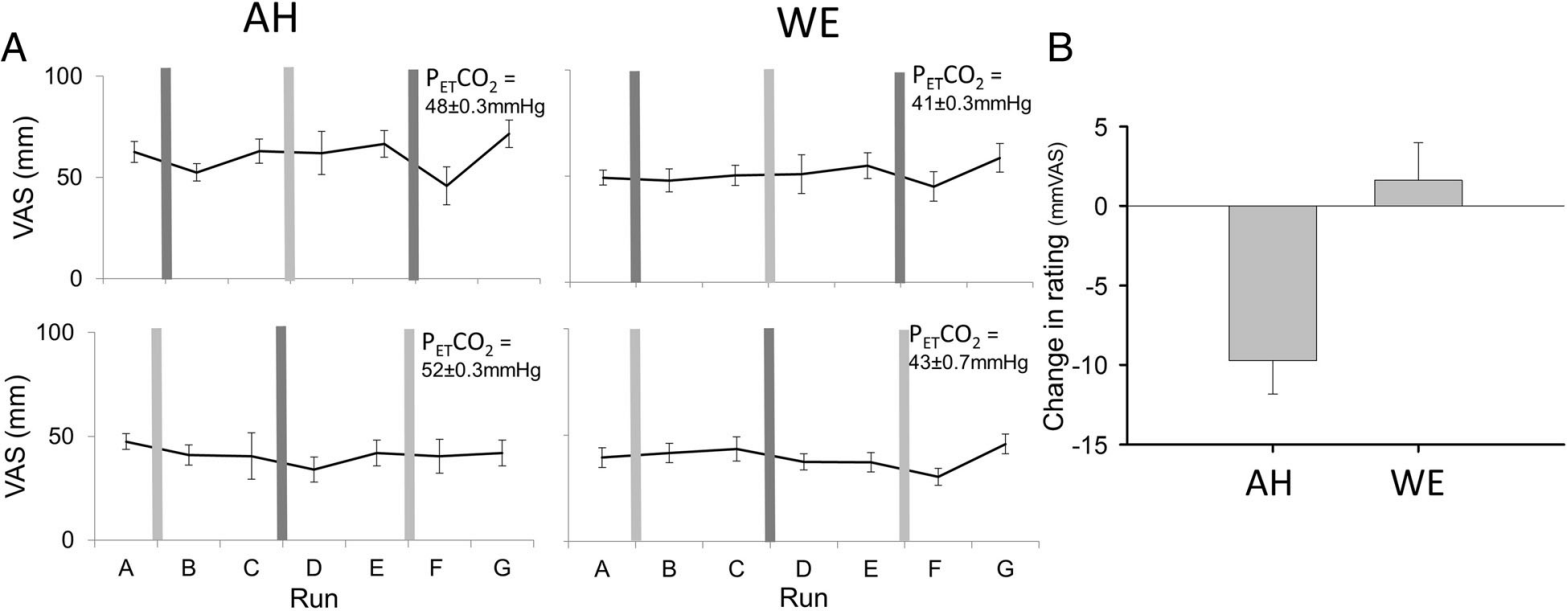
Overall changes in air hunger (AH) and work/effort (WE) associated with mist inhalations. Panel **a**. Mean ± SEM AH (left panels) and WE (right panels) before and after furosemide inhalations (black bars) and before and after saline inhalations (grey bars) in the 8 individuals who were allocated to the saline-furosemide-saline order of mist inhalations (top panels) and in 8 individuals who were allocated to the furosemide-saline-furosemide order of mist inhalations (bottom panels). VAS ratings improved to a greater extent after furosemide compared to saline mist inhalations for AH, but this pattern was not evident for WE. ETCO2 = end tidal CO2 (mean ± SD mmHg). Panel **b**. Least Squares Mean change in VAS ratings before and after inhaled furosemide relative to the change before and after inhaled saline for AH and WE

**Fig. 6:**
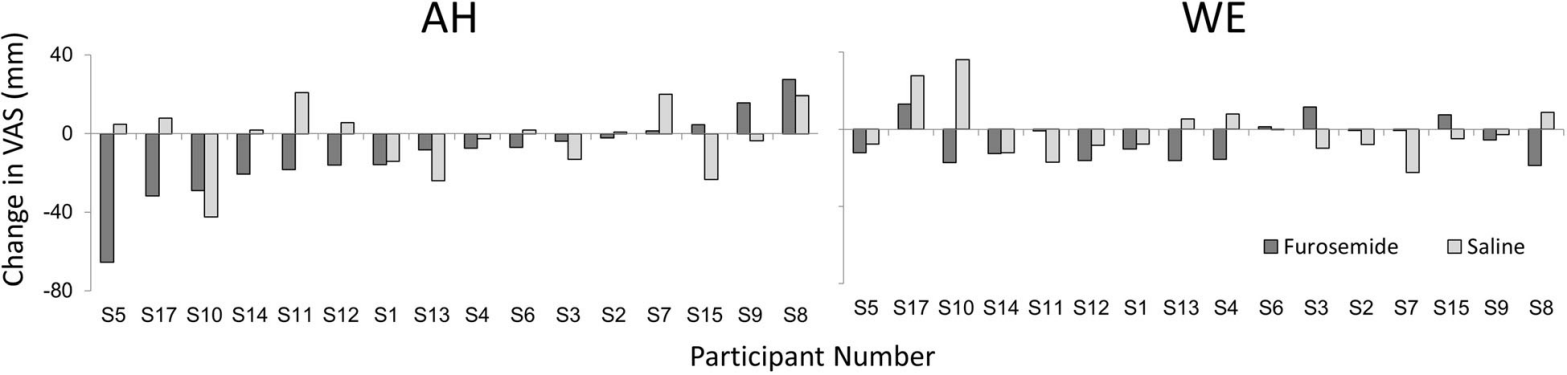
Individual data for change in visual analogue scale for air hunger (AH) and work/effort (WE). Individual change in visual analogue scale ratings (VAS, % full scale) of AH at fixed test levels of PETCO_2_ (left panel), and of the sense of breathing WE at fixed test levels of tidal volume (right panel) following inhaled furosemide (dark bars) and inhaled saline (grey bars). Closed bars indicate the average change in VAS for two furosemide inhalations in half the participants (S1, 2, 4, 7, 10, 11, 14, 17) or the change in VAS for one furosemide inhalation in the other half. Open bars indicate the average change in VAS for two saline inhalations in half the participants (S3, 5, 6, 8, 9, 12, 13, 15) or the change in VAS for one saline inhalation in the other half. Inhalation of furosemide tends to produce a reduction in VAS after furosemide more often than after saline for the AH test. For WE test reductions were evident for both inhaled furosemide and inhaled saline. Participants are arranged in order of response to furosemide for the AH test

A treatment effect (relief with inhaled furosemide relative to relief by inhaled saline control) was seen with furosemide for the AH test (Fig. 5). Mean VAS for dyspnoea was significantly lowered by furosemide relative to saline inhalation (Difference of Least Squares Mean ± SE of − 9.7 ± 2.1%VAS) for the AH test (*p* = 0.0015, Tukey-Kramer adjusted), but was not significantly changed by furosemide relative to saline inhalation (+ 1.6%VAS ± 2.4SE) for the WE test (*p* = 0.903, Tukey-Kramer adjusted). Four of the 16 participants showed a relief of over 20%VAS with inhaled furosemide for AH but no relief of this magnitude was seen in any participants for WE (Fig. 6).

### Single versus two doses of furosemide

**Fig. 7:**
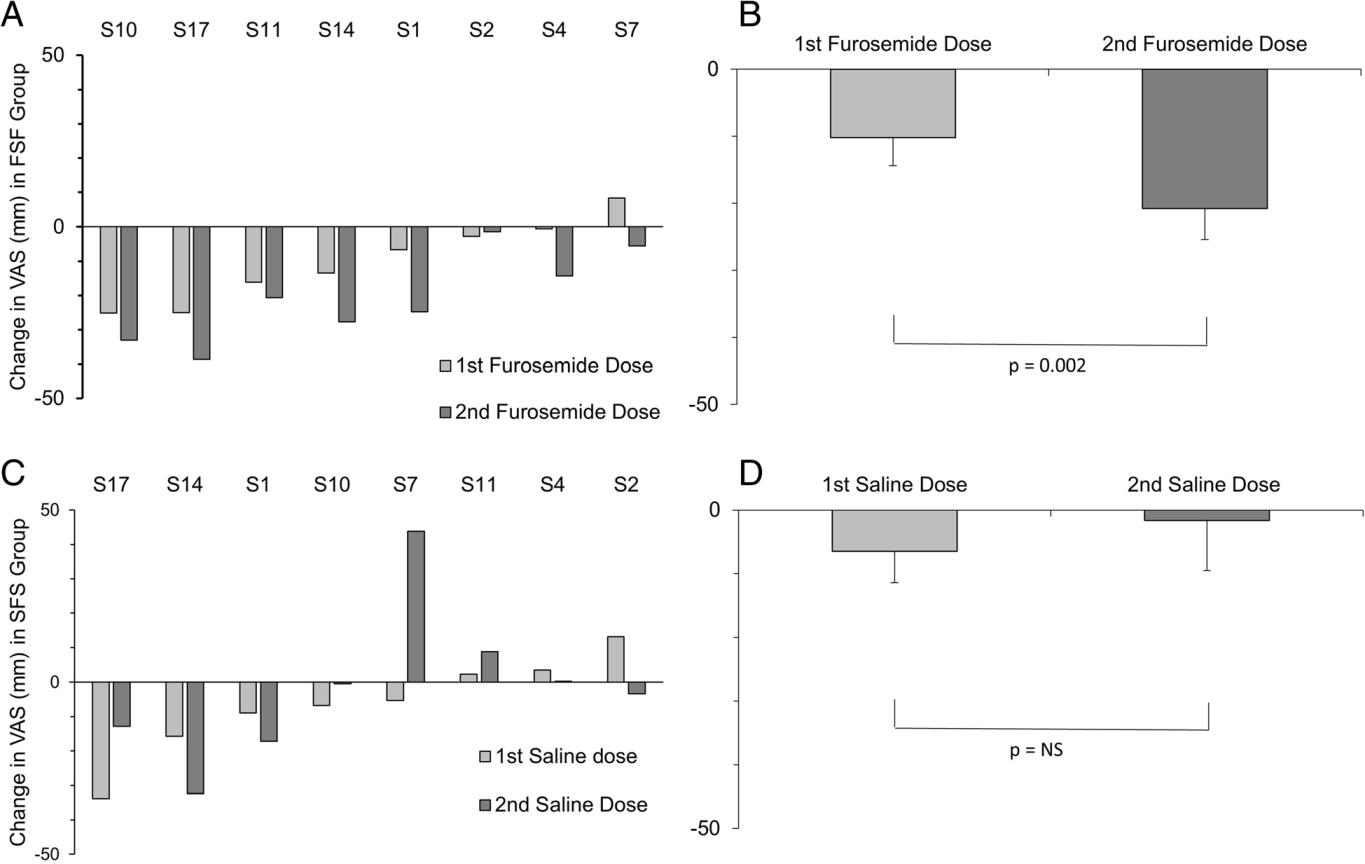
Second dose effect. Left panels (**a** and **c**): Individual (*n* = 8) changes in VAS ratings of AH in response to first and second doses of inhaled furosemide in the furosemide-saline-furosemide (FSF) group (**a**). Corresponding changes for the first and second doses of inhaled saline in the saline-furosemide-saline (SFS) group (**c**). For the FSF group the second dose of furosemide had a greater reduction in AH relief than the first dose in all but one participant. This was not true for the second dose of saline in the SFS group. Participants are arranged in order of response to first dose of furosemide for the FSF group or first dose of saline for SFS group. Right panels (**b** and **d**): The mean reduction in AH for the first and second dose of furosemide (**b**) and saline (**d**)

The average relief of AH from furosemide inhalations (averaged response for mists 1 and 3; 2x40mg) in the FSF group was greater than the relief seen with the single furosemide inhalation (mist 2; 1x40mg) in the SFS group; this group-wise comparison did not achieve statistical significance (mean ± SD − 15.5 ± 12 versus − 6.6 ± 27%VAS, unpaired t-test with unequal variance; *p* = 0.42). However, within the FSF group, 7 of the 8 participants had substantially greater relief of AH after the second inhalation (mist 3) of furosemide compared to the first (mist 1) - a doubling of relief (mean ± SD − 10 ± 12 versus − 21 ± 13%VAS) which was highly significant (paired t-test, *p* = 0.002; Fig. 7). In contrast, comparing the mean change for WE between the first and second doses of furosemide in the FSF group revealed no significant difference (paired t-test, *p* = 0.41). There were no significant differences between the first and second dose of saline within the SFS group for either AH or WE tests (paired t-test *p* = 0.6 and 0.3 respectively).

### Distinguishability of stimuli and blinding of participants

**Fig. 8:**
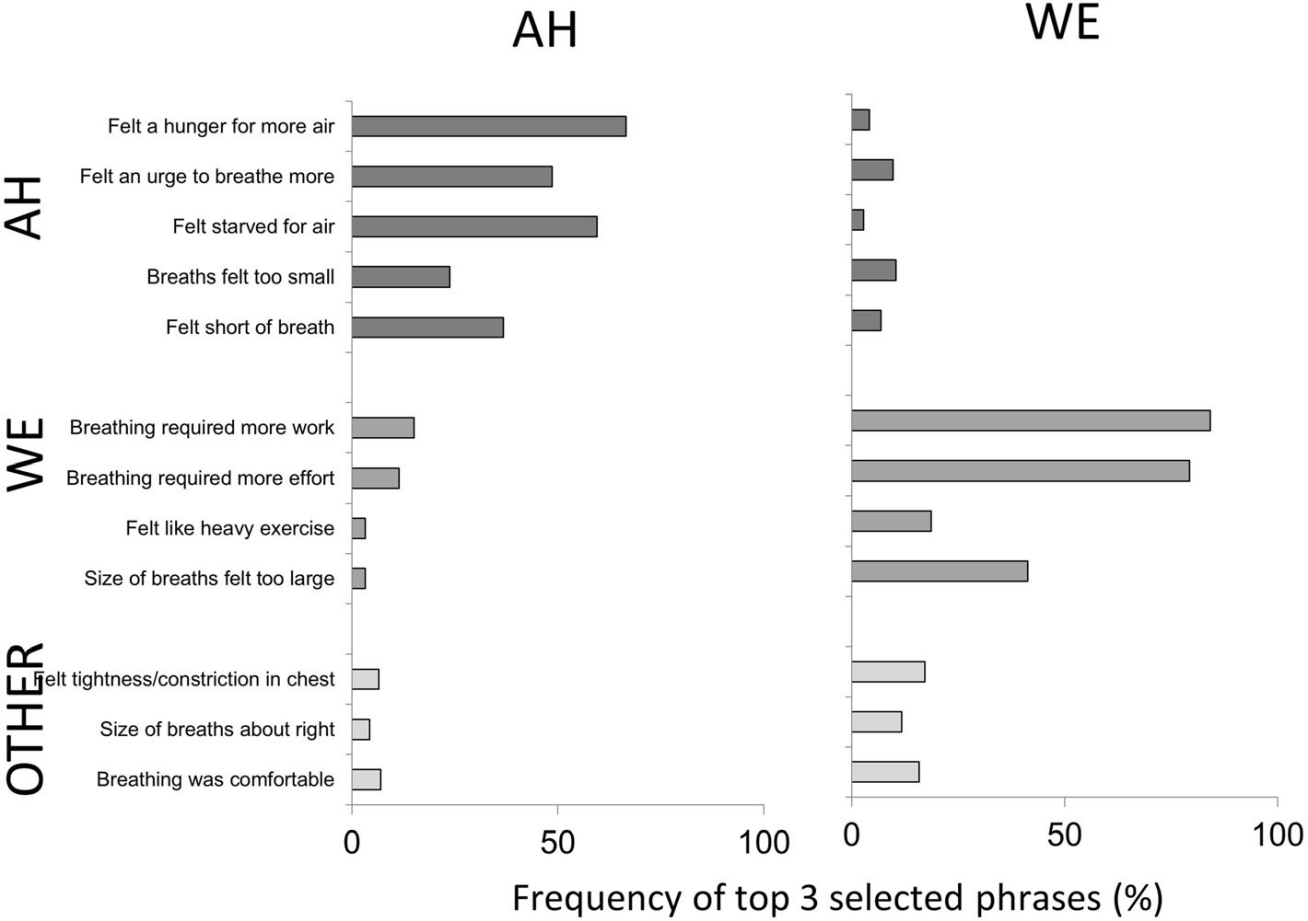
Dyspnoea Descriptors. Frequency with which each descriptive phrase was selected by participants to describe their experience during air hunger tests (AH; left panel) and WE tests (WE; right panel). AH cluster of descriptors dominated the participants’ choice of the respiratory sensations felt during the AH tests while the WE cluster of descriptors dominated the participants’ choice of the respiratory sensations felt during the WE tests

AH and WE stimuli were clearly distinguishable; subjective selection of descriptive phrases from a set list immediately following breathing tests verified that the AH test predominantly elicited phrases consistent with ‘air hunger’ whereas the WE test predominantly elicited phrases consistent with ‘work/effort’ (Fig. 8). Choice of descriptors following AH and WE test showed a low level of conflation in sensation ratings, with 6% choosing WE descriptors for the AH test and 10% choosing AH descriptors for the WE test.

Cumulative urine output was matched for FSF and SFS groups. There was no significant difference in the cumulative urine volume between participants in the FSF versus the SFS group with their concomitant intravenous infusions (mean ± SD, 1.6 ± 0.4 l versus 1.5 ± 0.5 l; *p* = 0.4). No other side effects related to the furosemide or saline inhalation were reported.

## Discussion

This study verifies that experimentally induced AH in healthy individuals is substantially relieved by inhaled furosemide compared to inhaled saline control. Furthermore, this study shows for the first time that this effect was specific for the AH component of dyspnoea rather than the sense of breathing effort.

### Confirmation of AH relief by inhaled furosemide

The same stimulus to generate AH (hypercapnia with constrained ventilation) and the same dose of inhaled furosemide, delivered by the same method was used in the Moosavi et al. (2007) study, which had demonstrated a borderline treatment effect with inhaled furosemide relative to saline inhalation [21]. The current study provides stronger evidence for a treatment effect with more robust data and was powered to support a definitive outcome.

In contrast to the current findings, Banzett et al. (2017) have recently published a study using similar methods in 11 healthy volunteers indicating no significant difference between relief of breathing discomfort by inhaled furosemide and by inhaled saline [22]. The effect of inhaled furosemide reported by Banzett et al. [22] was greater than ours (mean ± SE: − 17 ± 3 versus − 11 ± 5 %VAS); this is likely to reflect the fact that they used a higher dose (80 mg versus 40 mg) with controlled delivery on a mechanical ventilator. However, the effect of saline was far greater in the study by Banzett et al. [22] compared to this study (mean ± SE: − 13 ± 4 versus − 2.5 ± 4 %VAS); we believe this is likely to be due to an enhanced placebo effect in their study as participants were informed they were going to receive 2 active treatments and one saline, whereas in reality they had one active substance and two saline controls. Likewise, a second study published by this group [23] also reported a significant effect of inhaled saline (− 20 %VAS); this study also used a similar deception to that alluded above which is again likely to have enhanced the placebo effect. While this second study used the same dose (40 mg) to this current study they also reported a larger relief of ‘breathing discomfort’ with furosemide (− 20%VAS). This could be explained by the different delivery method used, which reduced loss of aerosol to the atmosphere during expiration [23]. The different delivery method they used reduces loss of aerosol to the atmosphere during expiration and also assuming a similar absorption efficiency (both studies in healthy volunteers) could have led to a greater and more prolonged interaction of furosemide with lung stretch receptors thereby accounting for a greater relief.

### Specificity of relief

The relief of experimentally induced dyspnoea in healthy individuals by inhaled furosemide was first shown by Nishino et al. (2000) who induced dyspnoea by i) a combination of inspiratory resistive load and hypercapnia, and ii) breath-holding [24]. The first of these stimuli was likely to have induced both WE and AH components of dyspnoea. Since participants were instructed to rate respiratory discomfort, both of these sensations could have contributed to their ratings. The breath hold task may have generated AH specifically but breath-holding is a non-steady state. A subsequent study in healthy individuals which specifically focused on experimentally induced steady state AH generated similar levels of relief [21]. This suggests that the inhaled furosemide may well have specifically relieved the AH component in the stimuli used by Nishino et al. [24].

In contrast there is direct evidence that inhaled furosemide does not affect the sensations associated with respiratory effort during expiratory flow limited exercise [25] or during exercise in the presence of external thoracic restriction in healthy individuals [26]. External thoracic restriction during exercise will elicit both AH and WE component of dyspnoea [26, 27]. In the study by Waskiw-Ford et al. (2018) individuals were asked to rate the intensity and unpleasantness of their perceived dyspnoea without specifying which component of dyspnoea to focus on [26]; it could be that the reported lack of relief was because of the participants focusing on WE due to the increased metabolic demand in this situation. Breathing effort is assumed to arise from non-vagal afferents from the chest wall, though a role for vagal afferents from the lungs in the sense of breathing effort cannot currently be discounted. Inhaled furosemide does appear to confer some benefit to exercising COPD patients but clinical dyspnoea in this scenario is likely to be multifactorial and may not be specifically related to respiratory effort [28]. It is generally accepted that AH and WE components of clinical dyspnoea likely arise from different neural pathways [8]. Previous reports have provided evidence that the AH component of dyspnoea is relieved by increased vagal afferent input from the lungs [7, 29–31] or accentuated by absence of vagal afferents from the lungs [32]. It is not known whether the vagal afferent information has any role in the sense of breathing effort - our data would suggest that vagal afferents have no role in generation of WE.

Inhaled furosemide demonstrated a statistically significant treatment effect that reached the accepted level for the minimally important clinical difference (MCID) for AH but not for WE [33]. There was some evidence of a placebo effect with, on average, a slight reduction in AH with inhaled saline. Some studies have shown a substantial relief of laboratory-induced dyspnoea with saline in over 30% of participants [22, 23]. However, O’Donnell and colleagues found that overall the administration of aerosol saline had little effect on experimentally induced AH, provided the expectation of a treatment effect is minimized [34]. In the current study, the careful blinding procedures and instructions participants received ensured that they were unable to guess correctly when they had received the active or placebo substance.

Ours is the first study to compare the effect of inhaled furosemide and inhaled saline on AH and WE induced separately in the same individuals allowing a direct comparison of treatment effects. Our data confirms that the mechanism of dyspnoea relief by inhaled furosemide, presumed to be via modulation of vagal afferents from the lungs, specifically relates to AH and not WE.

### Action of inhaled furosemide in the lungs

Inhaled furosemide is known to have many beneficial effects all of which appear to be mediated by actions on the airway epithelium such as; improvements in exercise induced asthma [35], inhibition of cough in asthmatics and healthy volunteers [36–38] and induced bronchodilation in constant-load exercise testing in COPD [39].

The most likely explanation for relief of dyspnoea with inhaled furosemide is modulation of lung mechanoreceptor feedback which replicates the sensation of larger tidal volumes; thus experimentally induced AH is relieved when tidal volume is increased [7, 40]. This is further supported by recent studies that have reported a weak to moderate correlation between the extent of dyspnoea relief with increased tidal volumes (whilst free breathing) on the one hand, and relief by inhaled furosemide on the other hand [22, 23]. Thus, inhaled furosemide may be acting at least in part via the same pathway.

There are many different mechanoreceptors in the lung including; slowly adapting pulmonary stretch receptors (saPSR), rapidly adapting pulmonary stretch receptors (raPSR), pulmonary and bronchial C-fibre receptors (irritant receptors). These receptors collectively transmit information to the central nervous system reporting the tidal volume or the presence of airway irritants [41] . Exposure of anaesthetized rats to inhaled furosemide has demonstrated sensitization of saPSRs and desensitization of raPSRs [6]. Evidence points to the saPSRs being the most likely mechanoreceptor involved since the raPSRs could not signal maintained volume changes as they provide feedback relating more to transition between inspiration and expiration rather than the magnitude of lung stretch [41, 42]. Studies in humans have suggested that it is feedback concerning the overall ventilation rather than intra-breath variables that account for the level of breathlessness perception [43, 44].

The diuretic effect of furosemide occurs via its chloride channel blocking property affecting the sodium-potassium-chloride co-transporter in the loop of Henle [5]. Because the same membrane co-transporters are expressed on vagal sensory neurons present in the airways [45] it is possible that the modulation of pulmonary stretch receptor sensitivity by inhaled furosemide may occur by the same mechanism. In vitro studies of isolated human lung tissue are needed to verify the precise mechanism of action of inhaled furosemide on pulmonary stretch receptors.

### No evidence for systemic action for dyspnoea relief with inhaled furosemide

A potential alternative mechanism of action of furosemide in relief of dyspnoea is via systemic effects from absorption of the inhaled furosemide into the circulation. Morélot-Panzini et al. (2018) estimated an absorption efficiency of up to 30% of the inhaled dose [23]. From this information and assuming the maximal level of efficiency, we estimated that a 40 mg inhaled dose would result in 5 mg entering the systemic circulation assuming a respiratory frequency of 12 breaths per minute and a duty cycle of 0.4. This appears to be higher than our estimate of systemic load of 1.5 mg from inhalation of a nebulized dose of 40 mg in this study. They also found that when given 15 mg intravenously participants had an average 16%VAS improvement in dyspnoea. It is suggested that in heart failure systemic furosemide relieves dyspnoea by easing pulmonary congestion and thereby reducing activation of pulmonary C-fibre receptors. [46, 47]. This cannot explain relief of dyspnoea by intravenous furosemide in healthy volunteers with no pulmonary congestion. The authors suggest that this result could be explained by the placebo effect as the participants were informed that they would only receive active substances. In the current study a dose of intravenous furosemide (1.5 mg) that more closely matched the amount absorbed from the inhaled dose was infused concurrently with inhaled saline. To maintain blinding intravenous saline was infused during furosemide inhalation. In both cases the rate of infusion was set to match the period of inhalation. We therefore consider the findings of the current study showing no significant relief from intravenous furosemide (mean ± SEM, − 2.5% ± 4) to demonstrate more clearly that the AH relief by inhaled furosemide is via direct actions within the lungs.

Further support for a mechanism of relief of AH via direct actions in the lungs is provided by: i) direct exposure of the lung tissue to furosemide in rat preparations resulted in modulation of PSR afferent activity but not when administered intravenously [6] ii) other studies in which beneficial effects of furosemide have been evident only when inhaled rather than administered via tablet [35] iii) absence of haemodynamic changes with inhaled furosemide in a study assessing wedge pressure measurements in heart failure patients suggesting no systemic mechanism of action [48] iv) absence of any detectable difference in cumulative urine output between the two groups in this study (SFS and FSF) which discounts a mechanism of action related to diuresis.

### Suggestion of ‘second dose’ effect

This study shows a beneficial cumulative effect of repeated furosemide inhalations (2x40mg). Ours is the only study that has to our knowledge investigated the effect of a second dose of inhaled furosemide on experimentally induced AH in healthy individuals. We noted a significant reduction in AH ratings with the second dose of furosemide in those who had the mists in the order FSF (mean ± SEM − 10.2 ± 4.2 versus − 20.8 ± 4.6%VAS). This was not seen for saline in those who had SFS so it is unlikely to be an order effect (mean ± SEM − 6.5 ± 5.0 versus − 1.6 ± 7.9). It is possible that the first dose of furosemide sensitizes the receptors so that the second dose has an additive/cumulative effect. Another possibility is from a carry-over effect where the inhaled furosemide is still active in the lungs for up to at least an hour after the first inhalation. Supporting this theory is Morélot-Panzini et al. (2018) study reporting that the rate of systemic absorption of inhaled furosemide is inversely related to the extent of dyspnoea relief [23]. This suggests that when the furosemide remains in the lungs, in contact with the pulmonary stretch receptors for a longer duration, the action of furosemide on dyspnoea relief is increased. An enhanced ‘second dose’ effect due to the pharmacokinetics is a recognized phenomenon in psychopharmocology [49].

The question remains whether the ‘second dose’ effect is related to sensitisation of stretch receptor-furosemide interaction or a carry-over effect from the first dose of furosemide due to incomplete removal of furosemide from the lungs before the second dose. In the current study the time between first furosemide mist and the second was approximately 90 min. If we accept that on average the furosemide stays within the lungs for up to 1 h based on the effect of a single dose on dyspnoea relief [21] the length of time between the first and second dose would go against a carryover effect to explain the greater relief with the second dose. This is further supported by the fact that in the SFS group the time between the middle furosemide mist and the second saline mist was less than 1 h and the pre second saline mist AH remained below the pre first saline mist level (consistent with a carryover effect of the middle furosemide mist). We would therefore favour a sensitisation explanation to account for the bigger relief from the second dose of furosemide in the FSF group.

### Technical considerations

Comparing AH versus WE tests it was difficult to achieve a target level of 50% VAS with the WE test. This resulted in the average VAS recordings for WE being lower (40-50mmVAS) compared to the AH average VAS recordings (50-60 mm VAS). A higher resistance in the circuit may have enabled both sensations to be studied at more comparable levels of the VAS. Though unlikely, we cannot discount the possibility that inhaled furosemide would not have been effective in relieving WE if the WE test had also been performed with a target level above 50% VAS.

It was noted that the time taken to nebulize 4 ml saline to 4 ml of furosemide differed (duration of saline mist was approximately 5-10 min and the furosemide approximately 10-15 min), and this was also reported independently in another recent study [23]. In the current study the unblinded researcher added saline or pretended to add a solution to the nebulizer to ensure the time taken to nebulize either solution was equal thereby maintaining blinding.

The participant selection of descriptive phrases after each breathing test confirmed that the different stimuli elicited the required sensations and that the participants were able to distinguish the different forms of dyspnoea (AH vs WE). For the AH tests patients were instructed to focus on and rate the form of dyspnoea indicated by the phrases they had previously selected following the initial exposure to the AH stimulus (during practice sessions). If the participant reported other sensations such as ‘breathing required more work’ during the AH tests, they were coached not to include this sensation in their ratings and to report them after each trial if present. For the WE tests the participants were instructed to focus on and rate the form of breathlessness indicated by the phrases they had previously selected following the initial exposure to the WE stimulus (during practice sessions). If the participant reported other sensations such as the AH descriptors, they were coached not to include this sensation in their ratings but to report them after each trial if present. After completing each trial, the participant described their sensations and picked phrases from a given list of descriptive phrases and identified the top 3 most relevant. Subject selections following the AH and WE tests were consistent with the type of stimulus. Participants were also queried about any non-respiratory sensations or external clues.

The participants and investigators were successfully blinded to the study drugs and no participant was able to correctly identify the correct order of mist inhalation. There was no detectable taste difference detected by the participants.

### Applicability of conclusions

This study was performed in a narrow age range (20-28 years). It is therefore not known whether the same results will apply to older population. It is possible that the sensitivity of PSRs alters with increasing age or is affected by lung/heart disease. Most patients with chronic dyspnoea will be much older than these study participants.

### Validity of conclusions

The test level of end tidal CO_2_ (ETCO_2_), the level chosen to generate 50% VAS full scale for AH at baseline, was different in the FSF group compared to the SFS group (48 ± 0.4 mmHg vs 52 ± 0.3 mmHg). We do not believe that this affects our data but it is interesting to consider why. A post prandial rise in ETCO_2_ has been demonstrated [50] but in our study there was no difference in consumption between the groups. They were also tested over both morning and afternoon sessions (FSF group: 3 in the morning, 5 in the afternoon. SFS group: 4 in the morning, 4 in the afternoon). There were no significant sex differences between the groups or differences in smoking habit. By chance there was an uneven distribution of ethnicity among the SFS and FSF groups (*p* = 0.031). All participants in the FSF group were Caucasian whereas the SFS group were not (Caucasian =3, Others = 5). There is some suggestion in the literature that the level of dyspnoea is associated with ethnicity [51]. This may explain some of the differences seen in this study. There was also a trend for increased weight (88 kg vs 70 kg) and for playing a wind instrument (3 vs 1) in the FSF group. In the SFS group more participants had a history of asthma (3 vs 0.) Although these were not statistically significant some of these differences in characteristics may explain the different ETCO_2_ levels in each group.

### Limitations

Ventilation, tidal volume and inspiratory reserve volumes were targeted at substantially different levels to generate AH and WE rated at approximately 50% on the VAS (9 vs 17 L/min; 0.75 vs 1.6 L, 1.8 vs 1.0 L respectively). For both AH and WE the levels of these variables were well matched before and after mist inhalations. However, the frequency of vagal feedback from PSRs will have been at a higher level for WE compared to AH test. We cannot therefore discount the possibility that had the WE test been done at the same level of afferent feedback from PSRs that the inhaled furosemide would have relieved WE as well. As discussed above (specificity of relief section) there is a lack of evidence for the role of vagal afferent feedback from PSRs in WE modulation. Furthermore from a practical viewpoint it would have been very difficult to strictly control the ventilatory parameters between AH and WE tests while maintaining a clear distinction in the quality of the dyspnoea generated and a far greater resistive load would have been required to generate 50% full scale on the VAS for WE.

In the VAS ratings of AH and WE we did not specifically ask patients to rate intensity or unpleasantness and it is likely that they rated a combination of both of these. From this study we cannot say which component was more predominant, however from previous studies we know that AH is more unpleasant than WE [22].

Since instructions prior to intervention could influence the outcome (e.g. amplify the placebo effects) we asked participants at the end of the study which order they thought they received the active and placebo substances. They were either unsure or chose an order that was not feasible (e.g. thought they received FFS or SSF or FSS etc.) We do not have any evidence that the small placebo effect we observed in this study arose from biasing the participant expectations through the instructions given prior to the start of the protocol.

## Conclusion

Inhaled furosemide was effective at relieving the AH component of dyspnoea but not the WE component. This is consistent with a mechanism involving sensitization of slowly adapting pulmonary stretch receptors leading to dyspnoea relief that specifically applies to the AH component, the most unpleasant form of dyspnoea. We suggest that multi-dimensional dyspnoea assessment tools should be used to identify patients where AH predominates the symptom burden and future clinical studies with inhaled furosemide should target these patients, irrespective of their underlying pathology, to optimise dyspnoea relief.

## Abbreviations

AH: Air hunger
COPD: Chronic Obstructive Pulmonary Disease
ETCO_2_: End tidal carbon dioxide
FSF: Furosemide-saline-furosemide (mist allocation order)
MCID: Minimally important clinical difference
NS: Not significant
PAW: Airway pressure
raPSR: Rapidly adapting pulmonary stretch receptors
saPSR: Slowly adapting pulmonary stretch receptors
SFS: Saline-furosemide-saline (mist allocation order)
VAS: Visual analogue scale
VT: Tidal volume
WE: Work/effort
